# Health facilities readiness for standard precautions to infection prevention and control in Nepal: A secondary analysis of Nepal Health Facility Survey 2021

**DOI:** 10.1371/journal.pone.0307589

**Published:** 2024-07-25

**Authors:** Bikram Adhikari, Ishwar Tiwari, Sulata Karki, Achyut Raj Pandey, Saugat Pratap K. C., Bipul Lamichhane, Shreeman Sharma, Suprich Sapkota, Bishnu Prasad Dulal, Ghanshyam Gautam, Deepak Joshi, Enrique Castro-Sánchez, Shyam Sundar Budhathoki, Sushil Chandra Baral

**Affiliations:** 1 HERD International, Lalitpur, Nepal; 2 University of Alberta, Alberta, Canada; 3 Imperial College London, Health Protection Research Unit in Healthcare-Associated Infection and Antimicrobial Resistance, London, United Kingdom; 4 University of the Balearic Islands, Global Health Research Group, Palma, Spain; 5 Brunel University London, College of Business, Arts, and Social Sciences, Uxbridge, United Kingdom; 6 Universidad Internacional de Valencia, Valencia, Spain; 7 Department of Primary Care and Public Health School of Public Health Imperial College London, London, United Kingdom; University College Dublin, IRELAND

## Abstract

**Background:**

Improvements in standard precaution related to infection prevention and control (IPC) at the national and local-level health facilities (HFs) are critical to ensuring patient’s safety, preventing healthcare-associated infections (HAIs), mitigating Antimicrobial Resistance (AMR), protecting health workers, and improving trust in HFs. This study aimed to assess HF’s readiness to implement standard precautions for IPC in Nepal.

**Methods:**

This study conducted a secondary analysis of the nationally-representative Nepal Health Facility Survey (NHFS) 2021 data and used the Service Availability and Readiness Assessment (SARA) Manual from the World Health Organization (WHO) to examine the HF’s readiness to implement standard precautions for IPC. The readiness score for IPC was calculated for eight service delivery domains based on the availability of eight tracer items: guidelines for standard precautions, latex gloves, soap and running water or alcohol-based hand rub, single use of standard disposal or auto-disable syringes, disinfectant, safe final disposal of sharps, safe final disposal of infectious wastes, and appropriate storage of infectious waste. We used simple and multiple linear regression and quantile regression models to examine the association of HF’s readiness with their characteristics. Results were presented as beta (β) coefficients and 95% confidence interval (95% CI).

**Results:**

The overall readiness scores of all HFs, federal/provincial hospitals, local HFs, and private hospitals were 59.9±15.6, 67.1±14.4, 59.6±15.6, and 62.6±15.5, respectively. Across all eight health service delivery domains, the HFs’ readiness for tuberculosis services was the lowest (57.8±20.0) and highest for delivery and newborn care services (67.1±15.6). The HFs performing quality assurance activities (β = 3.68; 95%CI: 1.84, 5.51), reviewing clients’ opinions (β = 6.66; 95%CI: 2.54, 10.77), and HFs with a monthly meeting (β = 3.28; 95%CI: 1.08, 5.49) had higher readiness scores. The HFs from Bagmati, Gandaki, Lumbini, Karnali and Sudurpaschim had readiness scores higher by 7.80 (95%CI: 5.24, 10.36), 7.73 (95%CI: 4.83, 10.62), 4.76 (95%CI: 2.00, 7.52), 9.40 (95%CI: 6.11, 12.68), and 3.77 (95%CI: 0.81, 6.74) compared to Koshi.

**Conclusion:**

The readiness of HFs to implement standard precautions was higher in HFs with quality assurance activities, monthly HF meetings, and mechanisms for reviewing clients’ opinions. Emphasizing quality assurance activities, implementing client feedback mechanisms, and promoting effective management practices in HFs with poor readiness can help to enhance IPC efforts.

## Introduction

Significant progress has been made in expanding healthcare coverage in low- and middle-income countries (LMICs) over the past few decades. However, the quality of care has not improved in accordance with progress in coverage. Quality of service demands that the services delivered are accessible, safe and effective, particularly in LMICs [1]. It is estimated that amenable death due to insufficient quality care would result in a substantial economic loss in LMICs, costing 2.6% of the gross domestic product compared to 0.9% in upper-middle-income countries [2]. The World Health Organization (WHO) estimated that each year, approximately 134 million adverse events occur in hospitals in LMICs, with unsafe care accounting for 2.6 million deaths alone [3, 4].

Healthcare associated infections (HAIs) are the most frequent adverse events, incurring a significant economic burden on healthcare systems worldwide and have a substantial impact on patients in terms of morbidity, mortality, and quality of life. The average prevalence of HAIs is 15.5% in LMICs, which is higher than in high-income countries (7.1% in Europe and 4.5% in the United States) [5]. In Nepal in 2022, the prevalence of HAI in tertiary care centers was 11% [6]. Most HAIs are preventable and can be reduced by up to 70% through implementing standard precautions for IPC measures [7]. For example, healthcare providers’ hands serve as the main vehicle for transmission, accounting for approximately 50% of HAIs, which can be prevented through standard precautions [8].

Centers for Disease Control and Prevention defined standard precautions as "the minimum infection prevention practices that apply to all patient care, regardless of suspected or confirmed infection status of the patient, in any setting where health care is delivered." [9]. The eight key elements of standard precautions related to IPC include performing hand hygiene; using personal protective equipment; following respiratory hygiene/cough etiquette principles; ensuring appropriate patient placement; cleaning and disinfecting patient care equipment, instruments/devices, and the environment; appropriate linen processing; following safe injection practices; and ensuring healthcare worker’s safety, including proper handling of needles and other sharps.

A global survey of 106 countries led by WHO in 2021–22 reported that an active IPC program existed in 54.7% of countries. The minimum requirement for IPC was met by only 3.8% of 106 countries [10]. Similarly, another global survey in 2019 reported that only 15.2% of the surveyed facilities (4440) fulfilled all the criteria established as the minimum requirements for IPC [11]. The minimum requirements for IPC are defined as IPC standards that should be in place at the national and facility level to provide minimum protection and safety to patients, healthcare workers, and visitors, based on the WHO core components for IPC programmes [10].

For effective management of IPC, it is recommended that national or subnational IPC programs be established, along with dedicated and well-trained IPC teams at the local and healthcare facility levels. In countries with limited IPC infrastructure, it is essential to assess the current IPC capabilities to identify areas requiring enhancement or development [12]. In Nepal, the Ministry of Health and Population (MoHP) launched the National IPC Guideline and shared the IPC implementation manual in 2022. This document supported and promoted the uniform implementation of IPC practices in healthcare facilities across the country [13], which is essential in achieving the third Sustainable Development Goal (SDG3) [14].

Studies assessing the readiness of health facilities (HFs) to implement standard precautions for IPC in Nepal’s context are scarce. A comprehensive assessment of the status of HF’s readiness is essential to strengthen the health system’s readiness to prevent HAI. A study has shown a negative relationship between HAI, the implementation of infection prevention and control measures, and the presence of essential components for infection prevention and control in HFs in Nepal [15]. Therefore, research on standard precaution for IPC practices in Nepalese HFs is essential to assess the safety of patients and healthcare workers. Thus, in this study, we examined the HF’s readiness to implement standard precautions to IPC, and its association with the characteristics of HFs using the secondary data from the NHFS 2021 [16].

## Methods

### Study design and setting

We analyzed secondary data from the Nepal Health Facility Survey (NHFS) 2021 [16]. NHFS 2021 is a nationally-representative cross-sectional survey carried out in both public and private HFs of Nepal [17]. Health services in Nepal are delivered by public, private, or other community-based or non-government organizations-run HFs, including clinics, medical centers, mission hospitals, or teaching hospitals. The public HFs are managed at three levels: federal, provincial, and local. The local health system includes primary hospitals, basic health service centers consisting of primary health care centers (PHCC), health posts, urban health clinics, community health units and outreach clinics at the community level designed to deliver constitutionally-mandated free basic health services. Health posts and basic health service centers are the first institutional contact point for basic health services. The federal and provincial-level health system includes central and provincial-level hospitals, mainly providing secondary and tertiary care. Each level above the health post is a referral point in a network ranging from PHCC to primary and tertiary-level hospitals. Private HFs, including private hospitals, polyclinics/clinics, medical halls, and pharmacies, complement public-sector health service delivery [17–19].

### Sample and sampling

The process of sample size estimation and sampling procedures involved in NHFS 2021 is explained elsewhere [17]. In brief, out of 5,681 HFs, 1,633 eligible HFs were selected in the NHFS 2021. The public HFs or private hospitals were eligible, whereas polyclinics or hospitals with stand-alone specialized services, such as care for cancer and heart conditions, were ineligible in the survey. The effective sample size of NHFS was 1626 after excluding eight duplicate HFs. The survey was completed in 1535 HFs, excluding stand-alone HIV Testing and Counseling Centers. The survey was not completed in the remaining HFs due to refusal, non-functional state of HF or unreachability. In this study, we analyzed data from 1535 HFs **(S1 Fig)**.

### Data collection

Data collection for NHFS 2021 took place between January 27 and September 28, 2021 [17]. The survey used a tool consisting of four types of survey instruments: a) Facility Inventory Questionnaire, b) Health Provider Questionnaire, c) Exit Interview Questionnaires, and d) Observation protocols for antenatal care, family planning services, care for sick children, and labour and delivery [17]. For this study, we used the data from the "Facility Inventory Questionnaire", which was done in both outpatient and inpatient settings.

The "Facility Inventory Questionnaire" was used to collect information from knowledgeable informants at facilities to determine whether facilities were ready to provide services at acceptable standards [17]. This questionnaire was standardized, validated, and tested across multiple countries by the "DHS program" [17]. The tool consisted yes/no questions assessing the availability of basic amenities for client services, basic equipments and supplies, capacity to perform basic laboratory tests, and availability of essential medicines defined by WHO. In addition, it assessed staffing levels, support systems for general management and quality assurance [17].

### Dependent variable

The dependent variable was the readiness score to implement standard precautions for IPC. In WHO’s Service Availability and Readiness Assessment (SARA) manual [20], the readiness score was calculated using nine tracer items. The nine tracer items included a) guidelines for standard precautions, b) latex gloves, c) soap and running water or alcohol-based hand rub, d) single-use disposable/auto-disable syringes, e) disinfectant, f) safe final disposal of sharps, g) safe final disposal of infectious wastes, h) appropriate storage of infectious waste, and i) appropriate storage of sharps waste. In this study, we did not use the ninth tracer item, an appropriate storage of sharp waste due to the unavailability of data. A tracer item, a medical mask, was used instead of single-use disposable/auto-disable syringes to calculate the readiness score for the tuberculosis service. The definition of tracer items is provided in the **S1 Table**.

Each HF’s readiness to implement safety precautions for IPC was captured through the eight service delivery domains: a) general outpatient care, b) child and adolescent vaccination services, c) child curative care, d) family planning, e) antenatal care services, f) delivery and newborn care, g) tuberculosis care and h) non-communicable care. Each tracer item was recorded as 0 or 1, 0 if the tracer item was not observed and 1 if observed. We calculated the readiness score for each service delivery domain by summing up eight tracer items divided by eight and multiplied by 100. The overall readiness score of HFs was obtained by averaging the scores of service delivery domains available in the health facility. The overall readiness score ranged from 0 to 100. The process of score calculation is illustrated in the **S2 Table**.

### Independent variables

The independent variables included location (rural/urban), ecological region (Hill/Mountain/Terai), province (Koshi/Madhesh/Bagmati/Gandaki/Lumbini/Karnali/Sudurpashchim), facility type (federal or provincial hospital/local HFs/private hospital), presence of external supervision (present/absent), quality assurance activities (performed/not performed) and frequency of health facility meeting (none/sometimes/monthly), and review of clients’ opinion (reviewed/not reviewed).

*Settings*. According to the Local Government Operation Act 2017, municipalities were classified into rural municipality, urban municipality, sub-metropolitan city and metropolitan city based on population, revenue generation, road connectivity, electricity and drinking water services [21]. Urban municipalities, sub-metropolitan cities and metropolitan cities were classified as urban or otherwise rural.

*Type of HFs*. The type of HFs was classified into federal or provincial hospitals, local HFs and private hospitals, where local HFs comprised local hospitals, health posts and primary health care centers.

*Quality assurance activities*. The facility was considered to have performed quality assurance activities if staff or members from the health facility reported carrying out quality assurance activities routinely and the interviewer observed documentation of a recent quality assurance activity, including report or minutes of a quality assurance meeting, a supervisory checklist, a mortality review, or an audit of records or registers [17].

*External supervision*. The facility was considered to have external supervision if facility staff or members reported receiving any external supervision/monitoring from the federal, provincial or municipal level in the past four months before the survey and the interviewer observed associated documentation [17].

*Review of clients’ opinion*. The HFs were considered to have reviewed clients’ opinions if staff or members of the health facility reported the presence of the system for determining clients’ opinion, a procedure for reviewing clients’ opinion, and the interviewer observed a report of a recent review of client opinion [17].

*Health facility meeting*. For frequency of health facility meetings, the HFs stating "no" for routine management/administrative meetings were classified as "None", those stating, "monthly or more often" were classified as "Monthly" and those stating, "irregular or every 2–6 months" were classified as "Sometimes" [17].

### Statistical analysis

We used R version 4.2.0 [22] and RStudio [23] for statistical analysis. We performed a weighted analysis to account for the complex survey design of NHFS 2021. Continuous variables were summarized using mean, Standard Deviation (SD), median and Interquartile Range (IQR). Frequency, percent (%), and (95% confidence interval (CI) around percent were used to summarize categorical variables. We employed simple and multiple linear regression analysis to examine the average effect of each predictor on the HF’s readiness score. We checked for the collinearity using the variance inflation factor. The selected variables in the model have a Variance Inflation Factor (VIF) less than 2.5 [24]. We applied a QR analytical approach to evaluate the association between different predictor variables and readiness scores with a set of quantiles ranging from 0.1 to 0.9. Compared with linear regression, QR extends to testing the effect of a predictor variable on an outcome variable at varying levels of the outcome variable rather than presuming a uniform mean effect [25].

## Results

### Characteristics of HFs

Of the 1535 HFs presented in Table 1, 1.8% were federal/provincial hospitals, 90.8% were local HFs, and 7.4% were private hospitals. Most of the HFs were from the hilly region (52.3%), followed by the Terai region (34.2%) and the Mountain region (13.4%). The highest proportion of HFs were from Bagmati province (20.5%) and the least from Karnali province (8.2%). Quality assurance activities were performed in 23.2% of overall HFs, the highest in federal/provincial hospitals (43.3%) and the lowest in private hospitals (18.9%). A review of clients’ opinions was performed in only 3.7% of total HFs. External supervision was carried out in more than 50%, and monthly HF meetings were carried out in at least 60% of all three types of HFs. All services were available in more than 90% of federal/provincial hospitals except child vaccination service (74.3%).

**Table 1:** Characteristics of HFs (n = 1535).

Characteristics of HF	Categories	All HFs % (95% CI)	Federal/Provincial hospitals, % (95% CI)	Local HFs, % (95% CI)	Private hospitals, % (95% CI)
Type of HFs	-	-	1.8 (1.4, 2.2)	90.8 (89.3, 92.1)	7.4 (6.2, 8.9)
Location	Urban	53.3 (49.6, 57.0)	95.9 (89.4, 98.5)	49.0 (45.0, 53.0)	96.1 (93.2, 97.8)
	Rural	46.7 (43.0, 50.4)	4.1 (1.5, 10.6)	51.0 (47.0, 55.0)	3.9 (2.2, 6.8)
Ecological region	Hill	52.3 (48.6, 56.0)	53.5 (43.4, 63.3)	52.6 (48.6, 56.6)	48.6 (40.0, 57.3)
	Mountain	13.4 (11.2, 16.0)	15.3 (9.4, 24.1)	14.1 (11.7, 17.0)	4.3 (1.7, 10.8)
	Terai	34.2 (30.7, 37.9)	31.2 (22.6, 41.3)	33.2 (29.4, 37.3)	47.1 (38.8, 55.6)
Province	Koshi	16.8 (14.1, 19.8)	16.4 (10.2, 25.3)	16.8 (13.9, 20.1)	16.7 (12.3, 22.2)
	Madhesh	15.7 (12.8, 19.2)	10.2 (5.5, 18.2)	16.1 (12.9, 19.9)	12.9 (9.3, 17.7)
	Bagmati	20.5 (17.7, 23.7)	20.5 (13.5, 29.8)	18.7 (15.7, 22.1)	43.1 (34.1, 52.6)
	Gandaki	12.6 (10.5, 15.1)	12.3 (7.0, 20.6)	12.9 (10.6, 15.6)	9.6 (6.7, 13.6)
	Lumbini	15.3 (12.9, 18.1)	16.1 (9.9, 25.2)	15.5 (12.8, 18.6)	12.8 (9.3, 17.4)
	Karnali	8.2 (6.7, 10.0)	11.3 (6.3, 19.4)	8.7 (7.0, 10.7)	1.7 (0.8, 3.6)
	Sudurpashchim	10.8 (9.1, 12.8)	13.3 (7.8, 21.8)	11.4 (9.5, 13.6)	3.1 (1.8, 5.4)
Quality assurance activities	Not performed	76.8 (73.4, 79.9)	56.7 (46.5, 66.3)	76.8 (73.1, 80.1)	81.1 (73.2, 87.0)
	Performed	23.2 (20.1, 26.6)	43.3 (33.7, 53.5)	23.2 (19.9, 26.9)	18.9 (13.0, 26.8)
External supervision	Absent	33.8 (30.4, 37.3)	27.7 (19.6, 37.6)	32.8 (29.2, 36.6)	47.2 (38.7, 55.8)
	Present	66.2 (62.7, 69.6)	72.3 (62.4, 80.4)	67.2 (63.4, 70.8)	52.8 (44.2, 61.3)
Review of client opinion	Not reviewed	96.3 (94.8, 97.3)	83.5 (74.5, 89.7)	97.4 (95.7, 98.4)	85.9 (78.9, 90.8)
	Reviewed	3.7 (2.7, 5.2)	16.5 (10.3, 25.5)	2.6 (1.6, 4.3)	14.1 (9.2, 21.1)
Health facility meeting	None	15.6 (13.2, 18.2)	7.2 (3.4, 14.4)	16.4 (13.9, 19.3)	6.8 (3.5, 12.8)
	Sometimes	20.5 (17.7, 23.6)	13.4 (7.8, 21.8)	20.9 (17.8, 24.2)	17.3 (11.9, 24.4)
	Monthly	60.0 (60.4, 67.4)	79.5 (70.1, 86.5)	62.7 (58.8, 66.4)	75.9 (68.0, 82.4)
Availability of services	General outpatient services	100.0	100.0	100.0	100.0
	Child and adolescent vaccination	88.9 (87.3, 90.4)	73.2 (63.6, 81.1)	93.2 (93.2, 95.5)	25.0 (18.3, 33.2)
	Child curative care	99.3 (99.0, 99.6)	99.0 (92.9, 99.9)	99.9 (99.6, 99.9)	93.1 (89.4, 95.6)
	Family planning	97.8 (97.0, 98.4)	95.9 (89.3, 98.5)	99.9 (99.8, 100)	71.8 (63.4, 78.9)
	Antenatal care services	98.3(97.6, 98.8)	96.9 (90.6, 99.0)	99.0 (98.4, 99.4)	90.0 (84.2, 93.8)
	Delivery and newborn care	51.4 (47.7, 55.1)	91.7 (84.1, 95.9)	50.5 (46.5, 54.5)	52.8 (44.1, 61.4)
	Tuberculosis care	79.9 (77.3, 82.3)	100.0	78.4 (75.5, 81.0)	94.1 (88.0, 97.2)
	Non-communicable care	96.9 (95.5, 97.9)	100.0	96.8 (95.3, 97.9)	97.3 (89.2, 99.4)

%: weighted percent; CI: confidence interval; n: weighted frequency; HFs: health facilities

### Distribution of IPC tracer items

**Fig 1:**
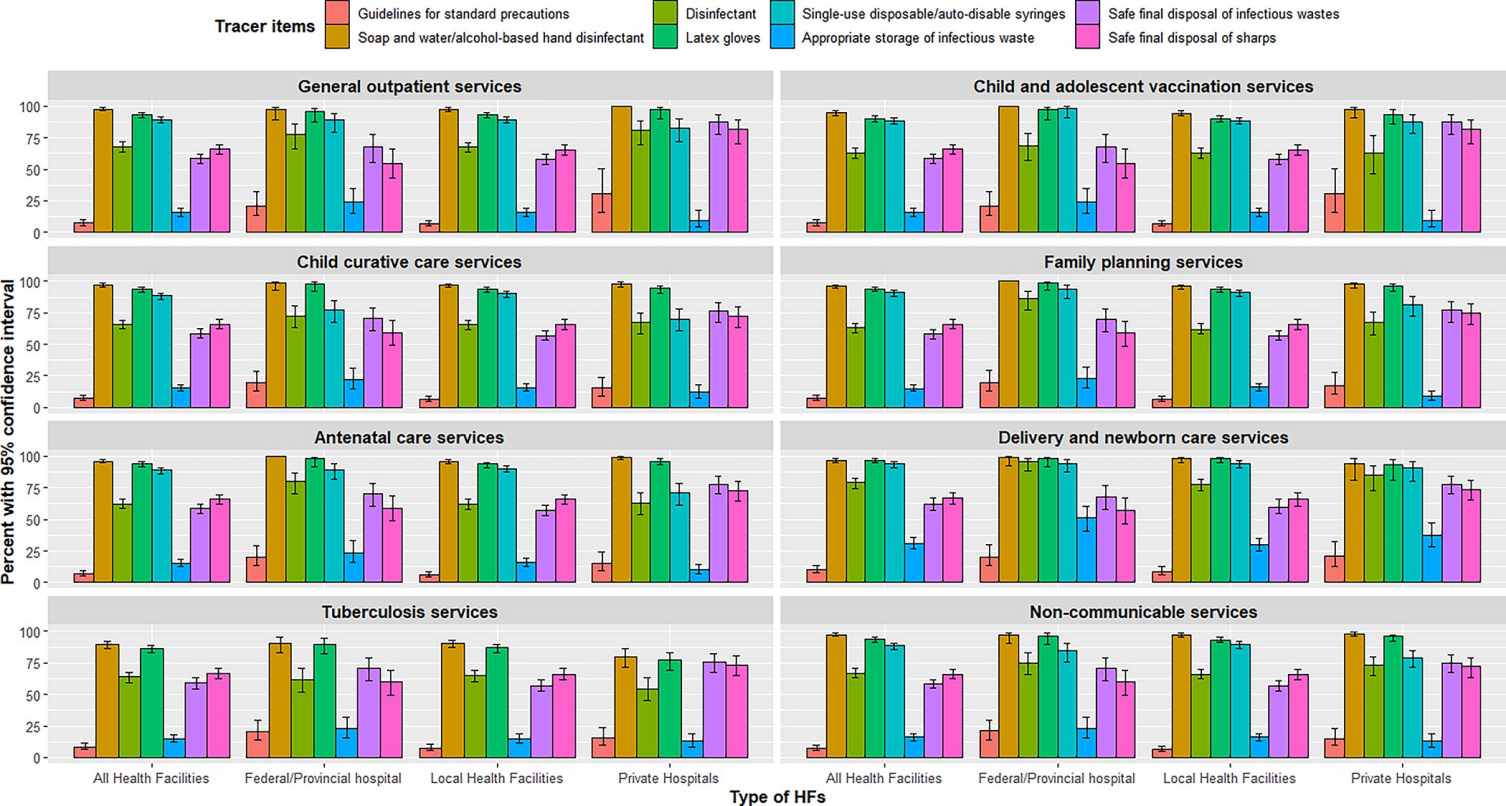
Distribution of IPC tracer items for different service delivery domains compared between federal/provincial hospitals, local HFs, and private hospitals.

We presented the distribution of IPC tracer items in each service delivery domain, comparing between different types of HFs (Fig 1). The guideline for standard precautions is the weakest domain, followed by medical waste disposal in each service delivery domain and each type of health facility. The percent and 95% CI of each tracer item by service delivery domain are presented in the S1 Fig.

### Readiness score of HFs to standard precaution for IPC

The HF’s readiness score is presented in Table 2. The overall readiness score of all HFs was 59.9±15.6. The overall readiness scores of federal/provincial hospitals, local HFs and private hospitals were 67.1±14.4, 59.6±15.6, and 62.6±15.5, respectively. The HFs readiness for tuberculosis service was the lowest (57.8±20.0), whereas all other services had readiness scores greater than 60.

**Table 2:** Service area-wise readiness score of HFs for standard precaution infection prevention and control.

Service area	All HFs		Federal/Provincial hospitals		Local HFs		Private hospitals	
	mean±SD	95% CI	mean±SD	95% CI	mean±SD	95% CI	mean±SD	95% CI
General outpatient services	61.9±16.2	60.7, 63.1	66.7±17.0	63.3, 70.1	61.4±16.1	60.2, 62.7	66.4±16.0	64.0, 68.8
Child and adolescent vaccination	60.5±17.1	59.1, 61.9	66.6±14.2	63.4, 69.9	60.2±17.1	58.7, 61.6	68.7±15.4	64.3, 73.0
Child curative care	61.6±16.0	60.4, 62.9	64.6±16.1	61.1, 68.0	61.5±16.0	60.2, 62.8	64.3±15.7	60.8, 67.8
Family planning	61.4±16.2	60.2, 62.6	68.8±14.5	65.9, 71.7	61.0±16.2	59.7, 62.3	65.1±15.8	62.0, 68.2
Antenatal Care (ANC) service area	61.1±16.0	59.9, 62.3	67.5±14.9	64.5, 70.5	60.8±16.0	59.5, 62.1	63.2±15.8	60.4, 66.0
Delivery and newborn care area	67.1±15.6	65.5, 68.7	72.9±15.4	69.7, 76.1	66.5±15.3	64.8, 68.3	71.7±17.5	67.1, 76.3
Tuberculosis care area	57.8±20.0	56.1, 59.5	62.9±19.1	58.1, 66.7	57.7±19.7	55.9, 59.6	57.2±21.8	53.1, 61.3
Non-communicable care area	61.8±16.2	60.6, 63.0	65.8±18.5	62.1, 69.5	61.5±16.1	60.2, 62.8	65.1±16.5	62.5, 67.7
Overall score of HFs	59.9±15.6	58.8, 61.1	67.1±14.4	64.2, 69.9	59.6±15.6	58.3, 60.8	62.6±15.5	60.2, 65.1

mean: weighted mean; SD: standard deviation; CI: confidence interval around mean; HFs: health facilities

### Factors associated with the readiness of HFs for standard precautions to IPC

The results from the simple and multiple linear regression, predicting the association between the HF’s readiness and predictor variables are presented in Table 3. In simple linear regression, the readiness score was significantly lower in the terai and mountain regions compared to hills. The HF’s readiness score was significantly higher in all provinces, except Madhesh, than in Koshi. Similarly, HFs with quality assurance activities and HFs reviewing clients’ opinions had significantly higher readiness scores than those that did not perform quality assurance activities and review of clients’ opinions.

**Table 3:** Simple and multiple linear regression model.

Variables	Readiness score, mean±SD	Simple linear regression		Multiple linear regression	
		β (95%CI)	p-value	β (95%CI)	p-value
**Type of HFs**					
Federal/provincial hospital	67.1±14.4	1		1	
Local HFs	59.6±15.6	**-7.49 (-13.44, -1.54)**	**0.014**	-4.93 (-10.77, 0.91)	0.098
Private hospital	62.6±15.5	-4.46 (-11.01, 2.09)	0.182	-3.85 (-10.23, 2.53)	0.237
**Location**					
Urban (ref)	60.2±16.4	1		1	
Rural	59.7±14.7	-0.53 (-2.10, 1.04)	0.507	-0.28 (-1.93, 1.37)	0.739
**Ecological region**					
Hill (ref)	61.7±15.5	1		1	
Mountain	58.4±15.4	**-3.26 (-5.64, -0.88)**	**0.007**	**-2.49 (-4.89, -0.08)**	**0.043**
Terai	57.9±15.7	**-3.72 (-5.43, -2.01)**	**<0.001**	0.25 (-2.07, 2.56)	0.836
**Province**					
Koshi (ref)	55.3±15.5	1		1	
Madhesh	55.3±16.2	0.07 (-2.61, 2.75)	0.957	-0.45 (-3.54, 2.65)	0.777
Bagmati	63.5±14.8	**8.22 (5.71, 10.74)**	**<0.001**	**7.80 (5.24, 10.36)**	**<0.001**
Gandaki	63.1±15.2	**7.79 (4.95, 10.64)**	**<0.001**	**7.73 (4.83, 10.62)**	**<0.001**
Lumbini	61.1±14.1	**5.88 (3.18, 8.59)**	**<0.001**	**4.76 (2.00, 7.52)**	**0.001**
Karnali	63.6±17.4	**8.37 (5.12, 11.63)**	**<0.001**	**9.40 (6.11, 12.68)**	**<0.001**
Sudurpashchim	59.1±14.3	**3.79 (0.81, 6.77)**	**0.013**	**3.77 (0.81, 6.74)**	**0.013**
**Quality assurance activities**					
Not Performed (ref)	58.9±15.8	1		1	
Performed	63.5±14.4	**4.61 (2.78, 6.45)**	**<0.001**	**3.68 (1.84, 5.51)**	**<0.001**
**External supervision**					
No (ref)	59.8±16.3	1		1	
Yes	60.0±15.3	0.28 (-1.37, 1.94)	0.738	-0.16 (-1.81, 1.48)	0.845
**System to take client opinion**					
No (ref)	59.6±15.6	1		1	
Yes	68.7±13.3	**9.09 (4.98, 13.19)**	**<0.001**	**6.66 (2.54, 10.77)**	**0.002**
**Frequency of HF meeting**					
None (ref)	57.1±15.5	1		1	
Sometimes	59.9±16.4	**2.76 (0.14, 5.38)**	**0.039**	**2.68 (0.12, 5.24)**	**0.040**
Monthly	60.7±15.3	**3.54 (1.34, 5.75)**	**0.002**	**3.28 (1.08, 5.49)**	**0.004**

β: beta coefficient; CI: confidence interval; OLS: ordinary least square regression; HFs: health facilities; ref: reference group

**Bold** indicates significance at 95% CI

In the multiple linear regression model, the HFs performing quality assurance activities had a 3.68 (95%CI: 1.84, 5.51) percent point higher readiness score and HFs with the system of reviewing clients’ opinions had a 6.66 (95%CI: 2.54, 10.77) percent point higher readiness score compared to those that did not perform quality assurance activities and did not have a system of reviewing clients’ opinions, respectively. The HFs with regular monthly meetings had a 3.28 (95%CI: 1.08, 5.49) percent point higher readiness score compared to HFs without HF meetings.

The HFs from Bagmati, Gandaki, Lumbini, Karnali and Sudurpashchim had readiness scores higher by 7.80 (95%CI: 5.24, 10.36), 7.73 (95%CI: 4.83, 10.62), 4.76 (95%CI: 2.00, 7.52), 9.40 (95%CI: 6.11, 12.68), and 3.77 (95%CI: 0.81, 6.74) percent point compared to Koshi.

**Fig 2:**
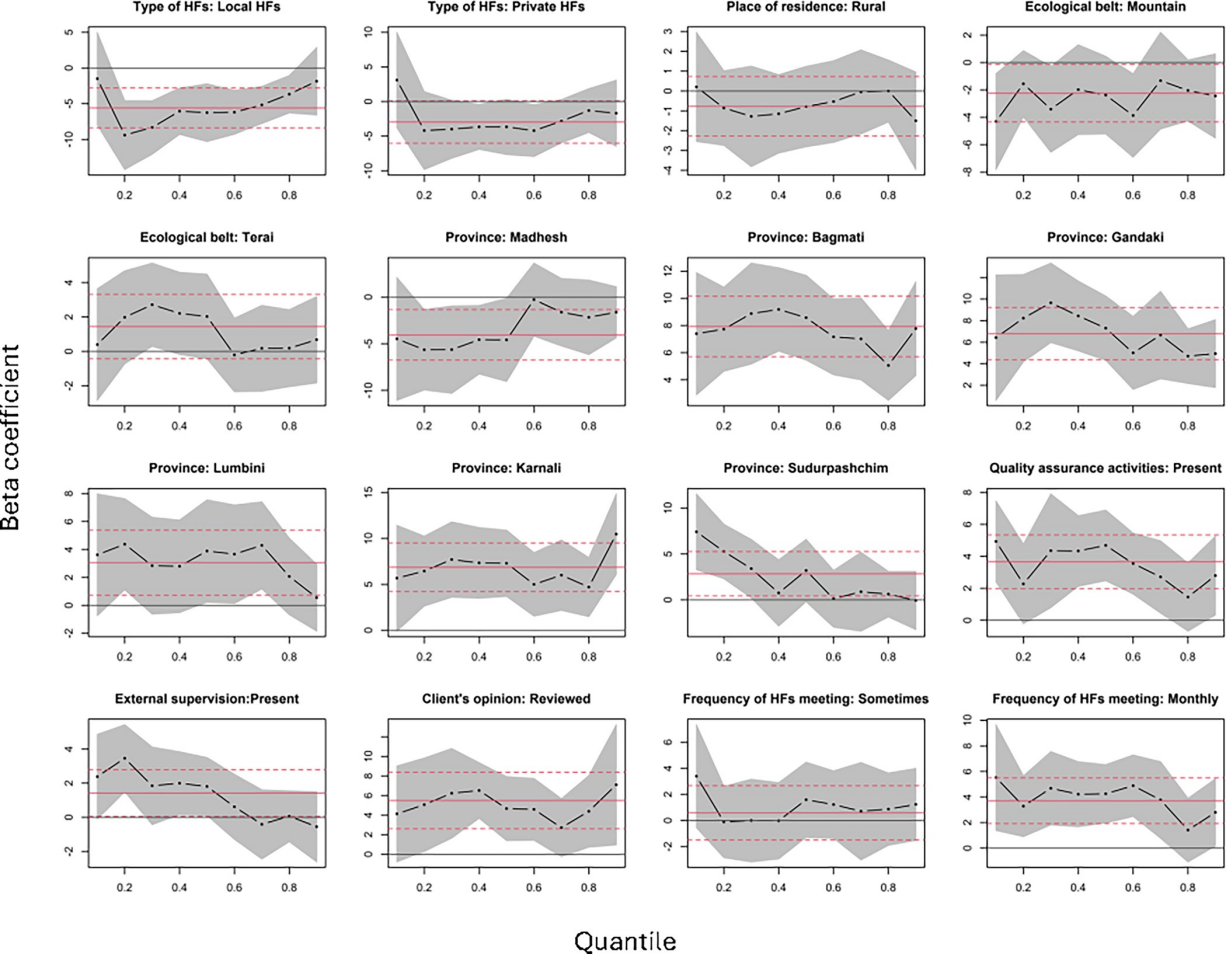
Quantile regressions predicting HF’s readiness score at 0.1 to 0.9 quantile. Each black dot is the slope coefficient for the quantile indicated on the x axis. The red lines are the least squares estimate and its confidence interval.

We presented the quantile levels of readiness score ranging from 0.1 to 0.9 on the x-axis and the regression coefficients for the associations of readiness score quantile levels with different predictors (β) derived from QR models on the y-axis (Fig 2). The 95% CI of the regression coefficients is also shown in Fig 2. The readiness score of local HFs was significantly lower than that of federal/provincial hospitals in all quantiles, ranging from 0.2 to 0.8 after adjusting for all other independent variables. After adjusting for all independent variables, the facilities with quality assurance activities had a higher readiness score at all quantiles between 0.3 and 0.7 and below 0.2. The facilities with the mechanism of reviewing clients’ opinions had a higher readiness score in quantiles ranging from 0.3 to 0.6 and above 0.7 after adjusting for all independent variables. Similarly, the facilities with regular monthly HF meetings had a higher readiness score in quantiles below 0.8 after adjusting for all independent variables.

## Discussion

This study examined Nepal’s HFs readiness to implement standard precautions for IPC and its association with the HF’s characteristics. In this study, the overall readiness score for HFs stood at around 60, with the highest readiness score for federal/provincial HFs (67) and the lowest readiness score for local HFs (60). In each type of HF and each service delivery domain, the guideline for standard precaution was the weakest domain. Across the eight service domains, the HFs readiness score was consistently lowest for tuberculosis service in all types of HFs. All types of HFs showed relatively higher readiness scores for delivery and newborn care services. The independent variables such as province, quality assurance activities, client opinions and frequency of health facility meetings showed statistically significant association with the HF’s readiness.

Federal/provincial HFs consistently demonstrated a greater readiness score than local HFs across quantiles ranging from 0.2 to 0.8. Similar to our findings, a study in Bangladesh reported that standard precautions for infection prevention components for lower-level facilities were lower than those for hospitals [26]. This disparity between higher-level and lower-level facilities may be attributed to the relatively greater access to resources, funding, and infrastructure in higher-level facilities as opposed to their lower-level counterparts. Despite these differences between higher and lower-level public HFs, it is imperative to enhance readiness in both types of facilities to mitigate the adverse impact of HAIs.

This study showed the guidelines for standard precaution as the weakest domain in each service domain and type of HF, indicating an urgent need for its improvement in Nepal. In line with this study, Hakim et al. [27] reported that guidelines for infection control were the least available in the HFs of Nepal compared to HFs from other LMICs like Afghanistan, Bangladesh, the Democratic Republic of the Congo, Haiti, Malawi, Senegal, and Tanzania. In Nepal, the IPC guideline was finalized only during the later phase of the COVID-19 pandemic in 2022 [15], lacking a proper guideline specific to IPC before 2022. The Infection Prevention or Health Care Waste Management Reference Manual 2014 [28] served as the only guideline for IPC before 2022. Therefore, the lack of a standard framework for IPC might contribute to the variation of readiness among countries [29]. A study by Tuladhar et al. [30] also reported low IPC implementation, with 7.9% for public and 8.4% for private HFs. The study highlighted the lesser availability of IPC guidelines despite the availability of trained providers on IPC and improved personal protective equipment in 2021, partly due to COVID-19 response efforts [30]. This could be due to logistic challenges or a gap in the supply chain of IPC guidelines. During the COVID-19 pandemic, the immediate focus was on equipping and training healthcare providers and ensuring PPE availability at the expense of a systematic rollout of comprehensive IPC guidelines, which could be another reason for the lesser availability of IPC guidelines [31].

This study showed that HFs offering tuberculosis services were least ready, indicating a higher risk of HAI through droplet infection. One study in Nepal reported that only 44% HFs have a general infection prevention plan, of which 69% had a tuberculosis infection control plan [32]. A study in Bangladesh also showed the lowest readiness of HFs for standard precaution while delivering tuberculosis services [26]. The inadequate readiness of HFs for standard precaution for tuberculosis service may be attributed to resource constraints, the burden of tuberculosis in the country and the corresponding burden on the health system, limited training and awareness opportunities, and inadequate infection control policies and implementation [12, 33]. There is a need for adequate preparedness to manage tuberculosis infections, specifically focusing on mitigating droplet transmission and reducing tuberculosis infections and other contagious diseases within healthcare settings [31].

HFs with quality assurance activities showed higher readiness scores, suggesting the importance of regular quality assurance activities in HFs in improving readiness for standard precautions. A low-quality assurance score suggests a faulty healthcare delivery system and a lack of preparedness to effectively prevent and control infectious outbreaks through a standardized approach [34]. Such a low-quality assurance may result in diminished client interest and satisfaction and increased mortality rates. Regular quality assurance activities enable HFs to assess their performance, identify improvement areas, and implement appropriate interventions to address deficiencies. This proactive approach helps to enhance the overall readiness of HFs to implement and maintain standard precautions, ultimately improving patient safety and preventing HAIs. This study revealed that facilities with good or moderate quality assurance achieved higher scores on IPC assessments. The significance of investing in quality assurance systems to substantially enhance adherence to infection prevention and control policies and guidelines is huge [35]. In alignment with our results, another study also found higher readiness scores in facilities with a client feedback system, thus demonstrating the importance of patient experiences and feedback in enhancing service delivery and quality [36].

This study has several strengths. First, we used data from a nationally representative survey of public and private HFs, allowing the generalizability of the findings for all HFs of Nepal. Second, we used weighted analysis to address complex survey design and non-response. However, this study is not free from limitations. The first limitation is that we could not analyze healthcare-provider-related factors in this study as the service provision assessment survey did not provide specific data on standard precautions for healthcare providers. Second, the cross-sectional nature of this study may mask situations where standard precautions were generally accessible but temporarily unavailable during the study or vice versa. Third, we have not assessed the presence of an active focal person or committee, which is essential for the effectiveness of the quality assurance system and for reviewing client opinions in improving the readiness of HF to implement standard precautions for IPC. Fourth, the survey was conducted during the COVID-19 pandemic, which could have positively influenced readiness scores [30].

## Conclusion

The HFs performing quality assurance activities, HFs with mechanisms for reviewing clients’ opinions and HFs from Bagmati, Gandaki, Lumbini, Karnali, and Sudurpashchim had a higher readiness to implement standard precautions. While the current readiness of HFs for standard precautions is promising, addressing identified areas like quality assurance, regular monthly meetings, reviewing clients’ opinion initiatives and promoting effective management practices could further improve the readiness of HFs for standard precautions to infection prevention and control in Nepal.

## Supporting information

S1 Checklist Human participants research checklist. (DOCX)

S1 Fig Sampling process and sample size. (PDF)

S1 Table Definition of each tracer items. (DOCX)

S2 Table Process of readiness score calculation. (DOCX)
