# Supplementary figures and images for: Health facilities readiness for standard precautions to infection prevention and control in Nepal: A secondary analysis of Nepal Health Facility Survey 2021
Source: PLoS One. 2024 Jul 25;19(7):e0307589. doi: 10.1371/journal.pone.0307589 (PMC11271867; doi:10.1371/journal.pone.0307589)

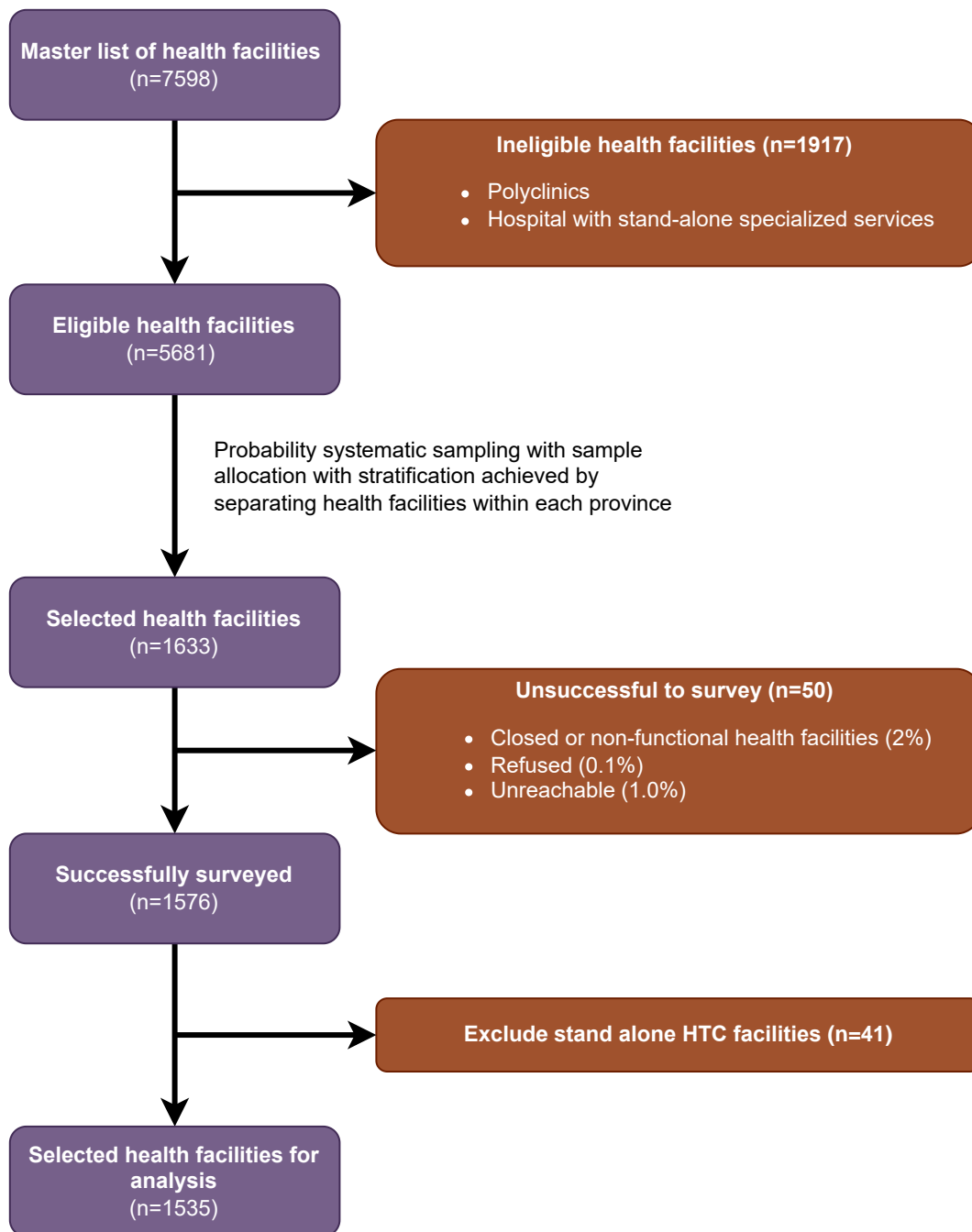

Supplementary Figure 1: Sampling process and sample size

Supplement: S1 Fig — (PDF) [file pone.0307589.s002.pdf]
