# Supplementary material for: Health facilities readiness for standard precautions to infection prevention and control in Nepal: A secondary analysis of Nepal Health Facility Survey 2021
Source: PLoS One. 2024 Jul 25;19(7):e0307589. doi: 10.1371/journal.pone.0307589 (PMC11271867; doi:10.1371/journal.pone.0307589)
Supplement: S1 Table — (DOCX) [file pone.0307589.s003.docx]

**Supplementary Table 1: Definition of each tracer items**

| **SN** | **Tracer item** | **Definition** |
| --- | --- | --- |
| T1 | Guidelines for standard precautions | The facility is considered to have availability of guideline for standard precautions if enumerator observed infection prevention and health care waste management reference manual 2015 or 2020. |
| T2 | Latex gloves | The facility is considered to have availability of latex gloves if enumerator observed latex gloves or non-latex gloves equivalent to latex gloves |
| T3 | Soap and running water or alcohol-based hand rub | The facility is considered to have availability of Soap and running water or alcohol-based hand rub if enumerator observed soap and running water or alcohol-based hand rub. |
| T4 | Single-use disposable/auto-disable syringes | The facility is considered to have availability of Single-use disposable/auto-disable syringes if enumerator observed single-use disposable or auto disable syringes. |
| T5 | Disinfectant | The facility is considered to have availability of disinfectants if enumerator observed any forms of disinfectants |
| T6 | Safe final disposal of sharps | The facility is considered to have availability of safe final disposal of sharps,  if enumerator observed the process of sharps waste disposal such as autoclave, or else the facility disposes of sharps waste by means of open burning in a protected area, dumping without burning in a protected area, burning and then dumping, or removal in the facility offsite with storage in a protected area prior to removal offsite. |
| T7 | Safe final disposal of infectious wastes | The facility is considered to have availability of Safe final disposal of infectious wastes if enumerator observed different process of health care waste (infectious waste other than sharps waste) disposal such as autoclave, or else the facility disposes of infectious waste by means of open burning in a protected area, dumping without burning in a protected area, burning and then dumping, or removal offsite with storage in a protected area prior to removal offsite. |
| T8 | Appropriate storage of infectious waste | The facility is considered to have availability of appropriate storage of infectious wastes if enumerator observed Waste receptacles. |
| T9 | Medical mask | The facility is considered to have availability of medical masks if enumerator observed surgical or N95 masks. |
