# Supplementary material for: Health facilities readiness for standard precautions to infection prevention and control in Nepal: A secondary analysis of Nepal Health Facility Survey 2021
Source: PLoS One. 2024 Jul 25;19(7):e0307589. doi: 10.1371/journal.pone.0307589 (PMC11271867; doi:10.1371/journal.pone.0307589)
Supplement: S2 Table — (DOCX) [file pone.0307589.s004.docx]

**Supplementary Table 2: Process of readiness score calculation**

**Step 1: All tracer items are coded as 1 or 0 based on their availability across all service delivery domains.**

| **SN** | **Tracer item** | **Score** | **Used in readiness score calculation of ____ Service delivery domain** |
| --- | --- | --- | --- |
| T1 | Guidelines for standard precautions | Available: 1  Not available: 0 | a, b, c, d, e, f, g, h |
| T2 | Latex gloves | Available: 1  Not available: 0 | a, b, c, d, e, f, g, h |
| T3 | Soap and running water or alcohol-based hand rub | Available: 1  Not available: 0 | a, b, c, d, e, f, g, h |
| T4 | Single-use disposable/auto-disable syringes | Available: 1  Not available: 0 | a, b, c, d, e, f, g, h |
| T5 | Disinfectant | Available: 1  Not available: 0 | a, b, c, d, e, f, g, h |
| T6 | Safe final disposal of sharps | Available: 1  Not available: 0 | a, b, c, d, e, f, g, h |
| T7 | Safe final disposal of infectious wastes | Available: 1  Not available: 0 | a, b, c, d, e, f, h |
| T8 | Appropriate storage of infectious waste | Available: 1  Not available: 0 | a, b, c, d, e, f, g, h |
| T9 | Medical mask | Available: 1  Not available: 0 | g |

*a) General outpatient care; b) Child and adolescent vaccination; c) Child curative care; d) Family planning; e) Antenatal care services ; f) Delivery and newborn care; g) Tuberculosis care; h) Non-communicable disease care*

**Step 2: Process of overall and service delivery domain wise readiness score calculation**

*a) Readiness score for General outpatient care (S1)= (T1+T2+T3+T4+T5+T6+T7+T8)/8*100*

*b) Readiness score for Child and adolescent vaccination (S2) = (T1+T2+T3+T4+T5+T6+T7+T8)/8*100*

*c) Readiness score for Child curative care (S3) = (T1+T2+T3+T4+T5+T6+T7+T8)/8*100*

*d) Readiness score for Family planning (S4) = (T1+T2+T3+T4+T5+T6+T7+T8)/8*100*

*e) Readiness score for Antenatal care services (S5) = (T1+T2+T3+T4+T5+T6+T7+T8)/8*100*

*f) Readiness score for Delivery and newborn care (S6) = (T1+T2+T3+T4+T5+T6+T7+T8)/8*100*

*g) Readiness score for Tuberculosis care (S7) = (T1+T2+T3+T4+T5+T6+T8+T9)/8*100*

*h) Readiness score for non-communicable disease care (S8) = (T1+T2+T3+T4+T5+T6+T7+T8)/8*100*

***Readiness score for IPC of HFs=(S1+S2+S3+S4+S5+S6+S7+S8)/8***

*Note: Appropriate storage of sharps waste, one of the tracer items is not used in readiness score calculations due to data unavailability*
